# Synergistic cell inactivation by cis-dichlorodiammineplatinum in combination with 1-propargyl-5-chloropyrimidin-2-one.

**DOI:** 10.1038/bjc.1987.189

**Published:** 1987-09

**Authors:** J. M. Dornish, E. O. Pettersen, R. Oftebro

**Affiliations:** Department of Tissue Culture, Institute for Cancer Research, Norwegian Radium Hospital, Oslo, Norway.

## Abstract

A synergistic effect with respect to inactivation of human NHIK 3025 cells cultured in vitro was displayed when treatment with cis-dichlorodiammineplatinum(II) (cis-DDP) and the mitotic inhibitor 1-propargyl-5-chloropyrimidin-2-one (NY 3170) were given in simultaneous combination. Cell inactivation was measured by loss of colony-forming ability. Treatment with NY 3170 alone produced no significant inactivation at concentrations up to 2 mM. However, treatment with NY 3170 in combination with cis-DDP induced increased cell inactivating effects equal to a doubling of either the concentration of cis-DDP or treatment time. Scheduling of NY 3170 treatment in relationship to a 1 h cis-DDP pulse revealed that synergism occurred only when the two drugs were present simultaneously. The inactivating effect of 10 microM cis-DDP in combination with 2 mM NY 3170 given to synchronized NHIK 3025 cells at various stages of the cell cycle was also determined. For cells treated in S or in G2 + M cell survival was reduced by a factor of 5 after a 1 h treatment with the drug combination as compared to similar treatment with cis-DDP alone. The cells appeared to be most sensitive at the time of initiation of DNA synthesis. Here cell survival was reduced by a factor of 100 following treatment with the drug combination than following treatment with cis-DDP alone. Measurement of cell-associated platinum by atomic absorption spectroscopy indicated that cellular uptake of cis-DDP was increased when NY 3170 was simultaneously present during drug treatment.


*Br. J. Cancer* (1987), **56**, 273–278 © The Macmillan Press Ltd., 1987

### Synergistic cell inactivation by *cis*-dichlorodiammineplatinum in combination with 1-propargyl-5-chloropyrimidin-2-one

J.M. Dornish, E.O. Pettersen & R. Oftebro

*Department of Tissue Culture, Institute for Cancer Research, The Norwegian Radium Hospital, Montebello, N-0310 Oslo 3, Norway.*

**Summary** A synergistic effect with respect to inactivation of human NHIK 3025 cells cultured *in vitro* was displayed when treatment with *cis*-dichlorodiammineplatinum(II) (*cis*-DDP) and the mitotic inhibitor 1-propargyl-5-chloropyrimidin-2-one (NY 3170) were given in simultaneous combination. Cell inactivation was measured by loss of colony-forming ability. Treatment with NY 3170 alone produced no significant inactivation at concentrations up to 2 mM. However, treatment with NY 3170 in combination with *cis*-DDP induced increased cell inactivating effects equal to a doubling of either the concentration of *cis*-DDP or treatment time. Scheduling of NY 3170 treatment in relationship to a 1 h *cis*-DDP pulse revealed that synergism occurred only when the two drugs were present simultaneously. The inactivating effect of 10 $\mu$M *cis*-DDP in combination with 2 mM NY 3170 given to synchronized NHIK 3025 cells at various stages of the cell cycle was also determined. For cells treated in S or in G2+M cell survival was reduced by a factor of 5 after a 1 h treatment with the drug combination as compared to similar treatment with *cis*-DDP alone. The cells appeared to be most sensitive at the time of initiation of DNA synthesis. Here cell survival was reduced by a factor of 100 following treatment with the drug combination than following treatment with *cis*-DDP alone. Measurement of cell-associated platinum by atomic absorption spectroscopy indicated that cellular uptake of *cis*-DDP was increased when NY 3170 was simultaneously present during drug treatment.

1-Propargyl-5-chloropyrimidin-2-one (NY 3170) is a mitotic inhibitor which causes reversible metaphase inhibition in cultured cells (Wibe & Oftebro, 1979). A sufficient, but necessary prerequisite for metaphase inhibition is that the drug be present during mitosis (Wibe *et al.*, 1979). The parent compound 5-chloropyrimidin-2-one (NY 3000) also induces metaphase arrest in cultured cells (Wibe *et al.*, 1978). Both NY 3170 and NY 3000 are members of a new class of mitotic inhibitors, called metahalones, which are based upon a pyrimidine structure (Gacek *et al.*, 1978). NY 3170 binds weakly to tubulin, the major protein of microtubules and the spindle apparatus (Dornish *et al.*, 1984*a*), and is a competitive inhibitor of colchicine binding to tubulin (Dornish & Oftebro, 1983; Dornish *et al.*, 1984*a*). The combination of NY 3170 and vincristine has shown more than additive effects with respect to cell inactivation when human cells of the cell line NHIK 3025 were treated with both agents (Wibe, 1980).

*Cis*-Dichlorodiammineplatinum(II) (*cis*-DDP) is known to act synergistically when administered in combination with various chemotherapeutic drugs (Gale *et al.*, 1977; Schabel *et al.*, 1979). Many pyrimidine compounds in combination with *cis*-DDP have demonstrated increased cell inactivating effects (Davidson *et al.*, 1975). Pyrimidine bases and nucleosides (Drewinko *et al.*, 1980), 1-$\beta$-D-arabinofuranosylcytosine (Bergerat *et al.*, 1981; Drewinko *et al.*, 1980; Schabel *et al.*, 1979) and 5-aza-2′-deoxycytidine (Vesely, 1982) in combination with *cis*-DDP have displayed marked synergistic effects both *in vivo* and *in vitro*. The exact mechanism responsible for synergism between pyrimidine compounds and *cis*-DDP is not known although Drewinko *et al.* (1980) have found an increase in the number of DNA cross-links following *cis*-DDP + 1-$\beta$-D-arabinofuranosylcytosine combination treatment.

This report presents results in which NY 3170 (a pyrimidine and a mitotic inhibitor) in combination with *cis*-DDP (a DNA damaging agent (Zwelling & Kohn, 1979; Zwelling & Kohn, 1980)) displays synergistic cell inactivation of human cells treated in culture.

Correspondence: J.M. Dornish.
Received 21 April 1987.

### Materials and methods

### *Cell line and cell synchronization*

NHIK 3025 cells originating from a human cervical carcinoma *in situ* (Nordbye & Oftebro, 1969; Oftebro & Nordbye, 1969) were cultivated as monolayers in medium E2a (Puck *et al.*, 1957) supplemented with 20% human and 10% horse serum (Gibco Europe Ltd., Renfrewshire, Scotland, UK). Under growth conditions used, the mean cell-cycle phase durations are 6.5 h for G1, 8 h for S and 2.5 h for G2. Mitosis lasts for 1 h. The median cell-cycle time is 18 h (Pettersen *et al.*, 1977). Cells were synchronized by mitotic selection (Pettersen *et al.*, 1977) and seeded immediately into 25 $cm^2$ plastic flasks (Falcon 3013, Falcon Plastics, Oxnard, CA, USA). All experiments involving synchronized cells were performed in a 37°C incubator room. The entrance of cells into the various cell-cycle phases was determined by flow cytometry of mithramycin-stained cells as described by Lindmo and Pettersen (1979) and Pettersen *et al.* (1983). Cell division was recorded in a separate 25 $cm^2$ flask where fields of ~100 cells were followed microscopically. The time of each cell division was recorded.

### *Cell survival*

The inactivating effect of drugs was determined using asynchronous NHIK 3025 cells seeded onto 60 × 15 mm style plastic petri dishes (Falcon 3002). A known number of cells was seeded in each of 5 replicate dishes and allowed to attach for 2 h. Drug-containing medium was then added and incubation continued for 1 h. Finally the drug-containing medium was removed, the dishes were rinsed with warm (37°C) Hanks' balanced salt solution and fresh medium was added. Incubation of cells for colony formation was for 12–14 days in a 5% $CO_2$ incubator holding 37°C. Medium was changed 7 days after the experiment was initiated. Colonies of cells were fixed in ethanol and stained with methylene blue. Colonies consisting of over 40 cells were counted.

For experiments involving synchronized cells, each data point represents counts from 4 replicate flasks. Cells were seeded into flasks immediately after mitotic selection and allowed to attach for 2 h before 1 h drug combination treatments were initiated at various times after mitotic selection. Cells were then washed with warm (37°C) PBS (NaCl 8,000 mg $l^{-1}$; $Na_2HPO_4 \cdot 2H_2O$ 1,150 mg $l^{-1}$; $KH_2PO_4$ 200 mg $l^{-1}$; KCl 200 mg $l^{-1}$) and fresh medium added. Incubation for colony formation was for 10–12 days at 37°C. Flasks were flushed with 5% $CO_2$ in air whenever opened. Colonies were fixed and stained as above.

*Atomic absorption spectroscopy*

Analysis of cell-associated platinum was performed using a Varian SpectrAA-30 atomic absorption spectrometer fitted with a GTA-96 graphite tube atomizer. Instrument control and data acquisition was by Varian Atomic Absorption Software. Automatic background correction with a modulated deuterium lamp was utilized. Cells were loosened from flasks by trypsin treatment and counted. A volume equivalent to $2 \times 10^6$ cells was pipetted into conical centrifuge tubes, three replicate tubes for each drug concentration. The tubes were centrifuged and cells were resuspended in drug-containing medium, usually 3 ml/tube. The cells were incubated with drugs at 37°C and held in suspension by using a rotary rack. After treatment the cells were centrifuged and washed in phosphate-buffered saline. The cell pellet was taken up into 100 $\mu$l concentrated $HNO_3$. Following overnight oxidation of organic material, 100 $\mu$l $H_2O$ was then added to each tube. Aliquots of 25 $\mu$l (representing 250,000 cells) were placed in a graphite tube and the atomic absorption signal measured at 265.9 nm following atomization of the sample was registered. Platinum content was quantitated by running a calibration curve immediately before the samples.

*Drugs*

*Cis*-dichlorodiammineplatinum(II) was purchased from Sigma Chemical Co., St. Louis, MO, USA. 5-Chloropyrimidin-2-one and 1-propargyl-5-chloropyrimidin-2-one (Figure 1) were provided by Prof. S. Laland, Department of Biochemistry, University of Oslo, Oslo, Norway. Drug solutions were made in phosphate-buffered saline or Hanks' balanced salt solution and sterile filtered. Drug combinations were mixed immediately before use using stock solutions diluted in medium E2a.

NY 3000 NY 3170

**Figure 1** Structure of 5-chloropyrimidin-2-one (NY 3000) and 1-propargyl-5-chloropyrimidin-2-one (NY 3170).

### Results

Figure 2 shows the surviving fraction of asynchronous (exponentially growing) NHIK 3025 cells following a 1 h treatment with *cis*-DDP or *cis*-DDP in combination with 2 mM NY 3170 as a function of the concentration of *cis*-DDP. NY 3170 alone has little or no effect on the survival of exponentially growing cells after such a short treatment period (Wibe & Oftebro, 1979). The data presented in Figure 2 indicate that the simultaneous presence of 2 mM NY 3170 resulted in an additional inactivating effect corresponding to that which a doubling of the *cis*-DDP concentration would give.

**Figure 2** Surviving fraction of asynchronous NHIK 3025 cells as a function of the concentration of *cis*-DDP (□) or *cis*-DDP + 2 mM NY 3170 (△). Single cells were plated on plastic dishes 2 h before medium with the appropriate concentration of drugs was added. Following a 1 h incubation period, the dishes were rinsed with warm (37°C) Hanks' balanced salt solution, then fresh medium was added. The experimental points represent the mean colony count from 5 replicate dishes. S.e. is represented by vertical bars when exceeding the size of the symbols.

To study how the increase in cell inactivation by *cis*-DDP depended upon the concentration of NY 3170, we performed another set of experiments where exponentially growing cells were treated with a combination of a fixed concentration of *cis*-DDP (either 6 or 10 $\mu$M) and NY 3170 at various concentrations. The results are presented in Figure 3 together with data showing the surviving fraction following treatment with NY 3170 alone. As demonstrated, cell inactivation induced by *cis*-DDP alone was significantly increased by the simultaneous presence of NY 3170.

As NY 3170 is synthesized from NY 3000 (Gacek *et al.*, 1979), the cell inactivating effect of a simultaneous combination of 6 or 10 $\mu$M *cis*-DDP and various concentrations of NY 3000 was also tested. The results in Figure 4 clearly show that the simultaneous presence of up to 5 mM NY 3000 did not have any effect on *cis*-DDP-induced cell inactivation. These results indicate the apparent importance of the 1-propargyl side group present in NY 3170 for *cis*-DDP + NY 3170 synergism.

The synergistic effect of NY 3170 and *cis*-DDP as a function of the duration of drug exposure is presented in Figure 5. In these experiments, NHIK 3025 cells were exposed to 10 $\mu$M *cis*-DDP either alone or in combination with 2 mM NY 3170 for up to 4 h. While treatment with 2 mM NY 3170 alone resulted in very small decreases in cell survival, treatment with 10 $\mu$M *cis*-DDP alone resulted in a survival which decreased with increasing drug exposure times. The simultaneous presence of 10 $\mu$M *cis*-DDP and 2 mM NY 3170 resulted in a large synergistic effect as compared with that after *cis*-DDP alone. From the data, 1 h treatment with 10 $\mu$M *cis*-DDP + 2 mM NY 3170 resulted in the same survival as 2 h treatment with 10 $\mu$M *cis*-DDP alone.

**Figure 3** Surviving fraction of asynchronous NHIK 3025 cells as a function of the concentration of NY 3170 (○), NY 3170+6 μM *cis*-DDP (▲), or NY 3170+10 μM *cis*-DDP (△). (■, □) represent 6 μM or 10 μM *cis*-DDP alone respectively. Experimental conditions and s.e. as in **Figure 2**.

**Figure 4** Surviving fraction of asynchronous NHIK 3025 cells as a function of the concentration of NY 3000 (○), NY 3000+6 μM *cis*-DDP (▲), or NY 3000+10 μM *cis*-DDP (△). (■, □) represent 6 μM or 10 μM *cis*-DDP alone respectively. Experimental conditions and s.e. as in **Figure 2**.

In the experiments described thus far *cis*-DDP and NY 3170 were added and removed simultaneously for combined treatment. We have also studied the combined effects when the treatment period for the two drugs were separated or overlapped only partially. The data in Figure 6 represents such an experiment where 10 μM *cis*-DDP was present for 1 h (horizontal line marking from 0 to 1 h) and NY 3170 (2 mM) was present as 1 h pulses either before, during, or after the *cis*-DDP treatment period. Survival is plotted as a function of the time when NY 3170 was added. Cells treated with 10 μM *cis*-DDP alone (squares) or with 2 mM NY 3170 alone (circles) are also shown.

**Figure 5** Surviving fraction of asynchronous NHIK 3025 cells as a function of the duration of drug treatment with 2 mM NY 3170 (○), 10 μM *cis*-DDP (□), and 2 mM NY 3170+10 μM *cis*-DDP (△). Single cells attached to plastic dishes were treated with each drug or drug combination for the time indicated in the figure. Drug treatment was terminated by removal of the drug-containing medium, thereafter cells were washed in Hanks' balanced salt solution and reincubated with fresh medium for colony formation. Data from 5 replicate dishes were averaged for each experimental point and s.e. is represented by vertical bars.

From Figure 6, NY 3170 (2 mM) given simultaneously with 10 μM *cis*-DDP resulted in a cell survival reduced to about one-tenth of that following treatment with *cis*-DDP alone. When NY 3170 was added 0.5 h later than *cis*-DDP and thus overlapped the pulse of *cis*-DDP by only 0.5 h, cell survival was now reduced by only one-half of that after *cis*-DDP alone. NY 3170 treatment immediately following the *cis*-DDP pulse resulted in identical survival as did treatment with *cis*-DDP alone. Further separation of NY 3170 treatment after *cis*-DDP resulted in no further change in survival.

Figure 6 also shows that the same is true when NY 3170 treatment precedes the *cis*-DDP pulse. Identical survival was measured until the treatment period for the two drugs overlapped by 0.5 h. When NY 3170 treatment preceded that of *cis*-DDP by 0.5 h, cell survival was again about one-half that following *cis*-DDP treatment alone. Maximum synergism with respect to cell inactivation thus occurs only when the two drugs are present simultaneously.

To determine whether the increase in *cis*-DDP-induced cell inactivation by NY 3170 was specific to any particular phase of the cell cycle, we treated synchronized cell populations with 1 h pulses of 10 μM *cis*-DDP alone or in combination with 2 mM NY 3170 at various times after mitotic selection. The results are shown in Figure 7. The synergistic effect of NY 3170 in combination with *cis*-DDP was present throughout the cell cycle. Cells in late G1/early S appeared to be the most sensitive where survival following treatment with *cis*-DDP+NY 3170 was reduced by a factor of 100 compared to that following *cis*-DDP treatment alone.

**Figure 6** Surviving fraction of asynchronous NHIK 3025 cells treated with 10 μM *cis*-DDP as a function of scheduling of 1 h pulses of 2 mM NY 3170 (△). (○) and (□) represent cell survival after a 1 h pulse of 2 mM NY 3170 alone or 10 μM *cis*-DDP alone respectively. The horizontal bar represents the treatment period for *cis*-DDP and the data points are plotted at the time at which drug incubation began. Each experimental point represents the mean of 5 replicate dishes. Vertical bars represent s.e.

**Figure 7** Surviving fraction of synchronized NHIK 3025 cells treated for 1 h with 2 mM NY 3170 (○), 10 μM *cis*-DDP (□), or 2 mM NY 3170 + 10 μM *cis*-DDP (△) as a function of the time after mitotic selection. Experimental points from 1 typical experiment are plotted at the time at which drug incubation was begun. The duration of the various cell-cycle phases for control cells is indicated at the bottom of the figure. Each experimental point represents the mean colony count from 4 replicate flasks. The vertical bars represent s.e.

Furthermore, a 1 h drug combination treatment in S and G2 resulted in a reduction of cell survival to about one-fifth of that following *cis*-DDP treatment alone.

Since the cell survival experiments indicated that treatment of cells with *cis*-DDP in combination with NY 3170 mimic a doubling of *cis*-DDP concentration, it was of interest to quantitate the platinum content in treated cells. Measurements of cell-associated platinum as performed by use of flameless atomic absorption spectroscopy are shown in Figure 8. The amount of cell-associated platinum in asynchronous cells treated for 1 h (open squares) or 2 h (filled squares) with *cis*-DDP increased as the concentration of *cis*-DDP in cell culture medium increased. The simultaneous presence of 2 mM NY 3170, however, caused an apparent doubling of the amount of cell-associated platinum. After a 1 h treatment with *cis*-DDP in combination with NY 3170 the amount of cell-associated platinum was the same as after a 2 h treatment with *cis*-DDP alone.

**Figure 8** The platinum content in NHIK 3025 cells measured by flameless atomic absorption spectroscopy as a function of the concentration of *cis*-DDP in cell culture medium. Cells were treated for 1 h (open symbols) or 2 h (filled symbols) with various concentrations of *cis*-DDP alone (□, ■) or in combination with 2 mM NY 3170 (△, ▲). The cells were then washed in PBS and organic material in the cell pellet was oxidized with $HNO_3$. Platinum content in an aliquot representing 250,000 cells was quantitated in triplicate for each drug concentration using the 265.9 nm absorbance line of platinum. Background correction utilizing a modulated deuterium lamp was applied. Vertical bars represent s.e.

### Discussion

The mitotic inhibitor 1-propargyl-5-chloropyrimidin-2-one previously shown to exert a synergistic effect in combination with vincristine (Wibe, 1980), also displayed synergism with respect to cell inactivation when NHIK 3025 human cells were treated with the drug in combination with *cis*-DDP (Figures 2, 3 and 5). Analysis of the data in Figure 2 reveals that the synergistic effect of NY 3170 is not of a dose-modifying type, but that it increases with increasing concentrations of *cis*-DDP. This is seen from Figure 2 by calculating the ratio between the *cis*-DDP concentrations in the absence and presence of NY 3170 at various survival levels. While this ratio is 1.6 at the survival level of 0.5, it is 2.0 at the survival level of 0.001. Thus, NY 3170 does not just increase the biological effects of *cis*-DDP by a certain factor, but its effect depends upon the *cis*-DDP concentration.

The maximal *cis*-DDP + NY 3170 synergism was found for 2 mM NY 3170 (Figure 3) even though this concentration of NY 3170 had no effect on cell inactivation alone. Higher concentrations of NY 3170 alone did, however, inactivate cells, thus 2 mM was the limit of this investigation. With respect to the duration of drug exposure, the presence of 2 mM NY 3170 together with *cis*-DDP caused similar cell survivals as would a doubling of the time of treatment with *cis*-DDP alone (Figure 5). Thus NY 3170 in combination with *cis*-DDP appears to cause cell inactivating effects that mimic either a doubling of *cis*-DDP concentration or treatment times.

The parent compound, 5-chloropyrimidin-2-one, also possessing metaphase inhibitory properties (Gacek *et al.*, 1979; Wibe *et al.*, 1978) did not display any effect on *cis*-DDP-induced cell inactivation (Figure 4). The only structural differences between these two molecules is the 1-propargyl group present in NY 3170 (see Figure 1).

Results from scheduling NY 3170 administration (Figure 6) show that NY 3170 must be present simultaneously with *cis*-DDP in order for the drug combination to exert a synergistic cell inactivating effect. When NY 3170 treatment either preceded or followed that of *cis*-DDP, cell survival was identical to that following treatment with *cis*-DDP alone. Slight overlapping of the two treatments resulted in a reduction in cell survival by about one-half for the drug combination than for *cis*-DDP alone. Simultaneous drug treatment resulted in a reduction of cell survival to about one-tenth of that following a 1 h pulse with *cis*-DDP alone.

From the above results two alternative assumptions are possible: (1) *cis*-DDP and NY 3170 could react to form a third compound of greater toxicity. (2) NY 3170 could increase cellular uptake of *cis*-DDP.

Regarding the first assumption we know that NY 3170 is able to penetrate cells quite readily (Wibe & Oftebro, 1981). Intracellular *cis*-DDP forms an aquated platinum species which react with nucleophilic sites (Zwelling & Kohn, 1979). The carbon–carbon triple bond of the propargyl group of NY 3170 may act as such a site. We have observed a reaction between *cis*-DDP and NY 3170 (data not shown), however, the kinetics of this reaction are too slow to explain the observed synergism between the two drugs. Nevertheless, we cannot rule out the possibility that just such a toxic reaction may be responsible for the large increase in cell inactivation seen when synchronized cells are treated in G1-phase.

From Figure 7 one can see that treatment of synchronized cells with *cis*-DDP alone caused an increased inactivation of those cells in early G1-phase. Combination treatment of *cis*-DDP together with NY 3170, however, caused a very large inactivation of cells in late G1-phase (i.e. from 6 to 8 h after mitotic selection) even though the two drugs were present for only 1 h. Perhaps this difference in cell-cycle specific inactivation may be due to just such a reaction between *cis*-DDP and NY 3170. We know that initiation of DNA synthesis in NHIK 3025 cells takes place from about 4 to 8 h after the end of mitosis (unpublished results). Although an unproven hypothesis, a possible reaction product between *cis*-DDP and NY 3170 *albeit* in small quantities, could block the initiation of DNA synthesis and cause irreversible damage to cells in which DNA synthesis was already initiated.

With respect to the second assumption we have previously shown that benzaldehyde and the vitamin $B_6$ aldehydes pyridoxal and pyridoxal 5′-phosphate modulate cellular toxicity of *cis*-DDP (Dornish *et al.*, 1984*b*, 1986; Dornish & Pettersen, 1985). In contrast to NY 3170, these aldehydes induced a protective effect in combination with *cis*-DDP. As it turned out, the protective effect was a result of reduced cellular uptake of *cis*-DDP due to the aldehydes. It is not known whether it is passive diffusion of *cis*-DDP (Gale *et al.*, 1973) or membrane-mediated transport (Byfield & Calabro-Jones, 1981), or both, that is influenced by the aldehydes. One can not exclude, however, that NY 3170 could have the opposite effect and increase cellular uptake of *cis*-DDP due to a membrane effect leading to enhanced cytotoxicity. Increased uptake of *cis*-DDP would also explain why NY 3170 potentiates the cell inactivating effect of *cis*-DDP in all stages of the cell cycle (Figure 7).

The results presented in Figure 8 confirm that, indeed, NY 3170 induces an increased uptake of *cis*-DDP into NHIK 3025 cells. Increased uptake of *cis*-DDP by NY 3170 is transient as shown by scheduling experiments in Figure 6 where the drugs must be in simultaneous combination. This could certainly be a membrane-mediated response to NY 3170. Finally, the structural moiety responsible for the synergistic effect must reside within the propargyl side group of NY 3170 since no such increase in *cis*-DDP uptake was seen when cells were treated with NY 3000 and *cis*-DDP (data not shown).

This study was supported by the Norwegian Cancer Society of which JMD is a Fellow. The Nansen Fund provided financial support for the Perkin-Elmer Model 3600 Data Station used for cell survival analysis. The Norwegian Research Council for Science and the Humanities (NAVF) provided funds for the Varian SpectrAA-30 atomic absorption spectrometer.

### References

BERGERAT, J.-P., DREWINKO, B., CORRY, P., BARLOGIE, B. & HO, D.H. (1981). Synergistic lethal effects of *cis*-dichlorodiammine-platinum and 1-$\beta$-D-arabinofuranosylcytosine. *Cancer Res.*, **42**, 25.

BYFIELD, J.E. & CALABRO-JONES, P.M. (1981). Carrier-dependent and carrier-independent transport of anti-cancer alkylating agents. *Nature*, **294**, 281.

DAVIDSON, J.P., FABER, P.J., FISCHER, R.G., Jr. & 4 others. (1975). 'Platinum-pyrimidine blues' and related complexes: A new class of potent antitumor agents. *Cancer Chemother. Rep.*, **59**, 287.

DORNISH, J.M. & OFTEBRO, R. (1983). Structure-activity relationship of 1-propargyl-5-halopyrimidin-2-ones. *Invest. New Drugs*, **1**, 203.

DORNISH, J.M., PETTERSEN, E.O. & OFTEBRO, R. (1984*a*). Interaction of 1-propargyl-5-chloropyrimidin-2-one (a metahalone) with rat brain tubulin. *Biochim. Biophys. Acta*, **797**, 156.

DORNISH, J.M., PETTERSEN, E.O., OFTEBRO, R. & MELVIK, J.E. (1984*b*). Reduction of *cis*-dichlorodiammineplatinum-induced cell inactivation by benzaldehyde. *Eur. J. Cancer Clin. Oncol.*, **20**, 1287.

DORNISH, J.M. & PETTERSEN, E.O. (1985). Protection from *cis*-dichlorodiammineplatinum-induced cell inactivation by aldehydes involves cell membrane amino groups. *Cancer Lett.*, **29**, 235.

DORNISH, J.M., MELVIK, J.E. & PETTERSEN, E.O. (1986). Reduced uptake of *cis*-dichlorodiammineplatinum by benzaldehyde. *Anticancer Res.*, **6**, 583.

DREWINKO, B., CORRY, P., BERGERAT, J.-P. & BARLOGIE, B. (1980). The lethal activity of platinum compounds in combination with pyrimidine derivatives. In *Cisplatin Current Status and New Developments*, Prestayko, A.W. *et al.* (eds) p. 37. Academic Press: New York.

GACEK, M., UNDHEIM, K., OFTEBRO, R. & LALAND, S.G. (1979). Metahalones, a new class of metaphase inhibitors. *FEBS Lett.*, **98**, 355.

GALE, G.R., MORRIS, C.R., ATKINS, L.M. & SMITH, A.B. (1973). Binding of an antitumor platinum compound to cells as influenced by physical factors and pharmacologically active agents. *Cancer Res.*, **33**, 813.

GALE, G.R., ATKINS, L.M., MEISCHEN, S.J., SMITH, A.B. & WALKER, E.M. (1977). Chemotherapy of advanced L1210 leukemia with platinum compounds in combination with other antitumor agents. *Cancer Treat Rep.*, **61**, 445.

LINDMO, T. & PETTERSEN, E.O. (1979). Delay of cell cycle progression after X-irradiation of synchronized populations of human cells (NHIK 3025) in culture. *Cell Tissue Kinet.*, **12**, 43.

NORDBYE, K. & OFTEBRO, R. (1969). Establishment of four new cell strains from human uterine cervix. I. *Exp. Cell Res.*, **58**, 458.

OFTEBRO, R. & NORDBYE, K. (1969). Establishment of four new cell strains from human uterine cervix. II. *Exp. Cell Res.*, **58**, 459.

PETTERSEN, E.O., BAKKE, O., LINDMO, T. & OFTEBRO, R. (1977). Cell cycle characteristics of synchronized and asynchronous populations of human cells and effect of cooling of selected mitotic cells. *Cell Tissue Kinet.*, **10**, 511.

PETTERSEN, E.O., NOME, O., RØNNING, Ø.W. & OFTEBRO, R. (1983). Effects of benzaldehyde on survival and cell cycle kinetics of human cells cultivated *in vitro*. *Eur. J. Cancer Clin. Oncol.*, **19**, 507.

PUCK, T.T., CIECIURA, S.J. & FISHER, H.W. (1957). Clonal growth *in vitro* of human cells with fibroblastic morphology. *J. Exp. Med.*, **106**, 145.

SCHABEL, F.M., Jr., TRADER, M.W., LASTER, W.R., Jr., CORBETT, T.H. & GRISWOLD, D.P., Jr. (1979). *cis*-Dichlorodiammineplatinum(II): Combination chemotherapy and cross-resistance studied with tumors of mice. *Cancer Treat. Rep.*, **63**, 1459.

VESELY, J. (1982). Synergistic effect of *cis*-dichlorodiammineplatinum and 5-aza-2′-deoxycytidine on mouse leukemic cells *in vivo* and *in vitro*. *Int. J. Cancer*, **29**, 81.

WIBE, E., OFTEBRO, R., CHRISTENSEN, T., LALAND, S.G., PETTERSEN, E.O. & LINDMO, T. (1978). Inhibitory effects of the new mitotic inhibitor 5-chloropyrimidin-2-one and of vincristine on human cells *in vitro*. *Cancer Res.*, **38**, 560.

WIBE, E. & OFTEBRO, R. (1979). Inactivation by the mitotic inhibitor NY 3170 of human cells *in vitro*. *Br. J. Cancer*, **40**, 222.

WIBE, E., OFTEBRO, R., LALAND, S.G., PETTERSEN, E.O. & LINDMO, T. (1979). Cell-cycle inhibitory effects of the mitotic inhibitor NY 3170 on human cells *in vitro*. *Br. J. Cancer*, **39**, 391.

WIBE, E. (1980). Age-dependent cell inactivation by vincristine alone or in combination with 1-propargyl-5-chloropyrimidine-2-one. *Cancer Res.*, **40**, 2069.

WIBE, E. & OFTEBRO, R. (1981). A study of factors related to the action of 1-propargyl-5-chloropyrimidin-2-one (NY 3170) and vincristine in human multicellular spheroids. *Eur. J. Cancer Clin. Oncol.*, **17**, 1053.

ZWELLING, L.A. & KOHN, K.W. (1979). Mechanism of action of *cis*-dichlorodiammineplatinum(II). *Cancer Treat. Rep.*, **63**, 1439.

ZWELLING, L.A. & KOHN, K.W. (1980). Effects of cisplatin on DNA and the possible relationships to cytotoxicity and mutagenicity in mammalian cells. In *Cisplatin Current Status and New Developments*, Prestayko, A.W. *et al.* (eds) p. 21. Academic Press: New York.


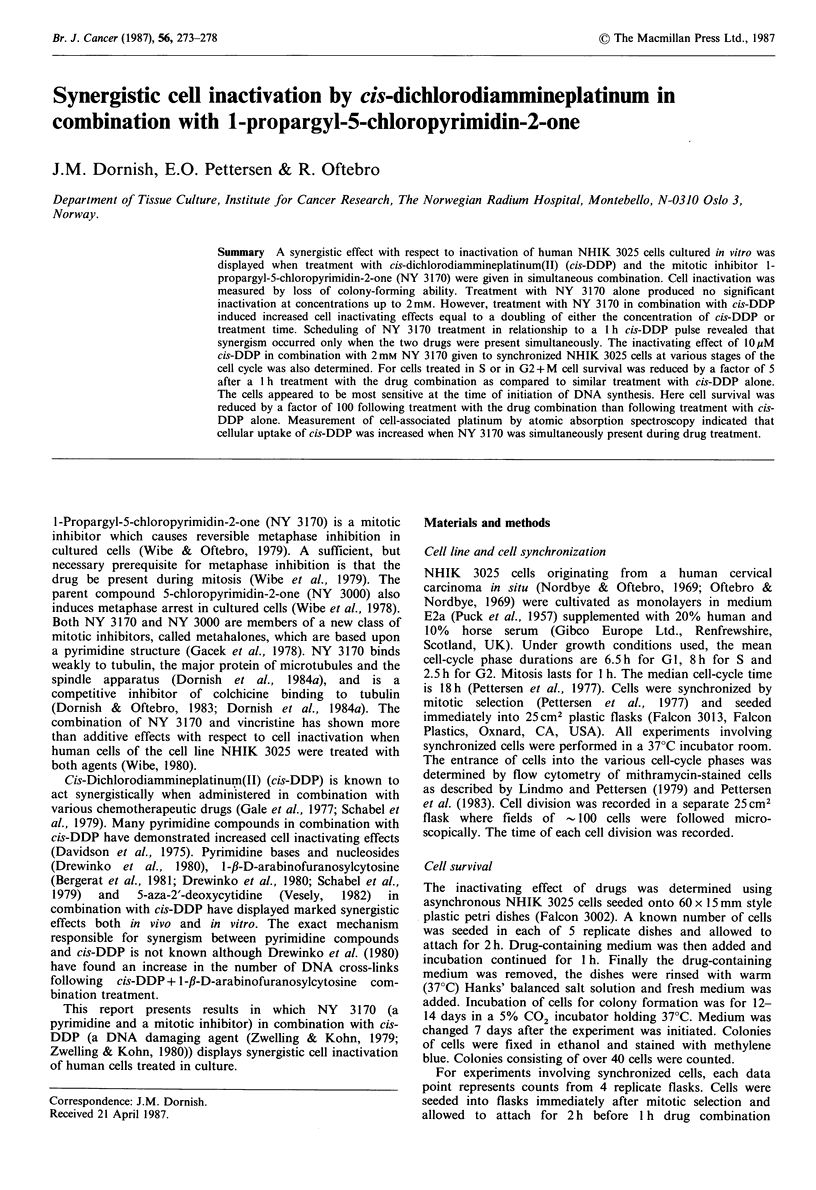

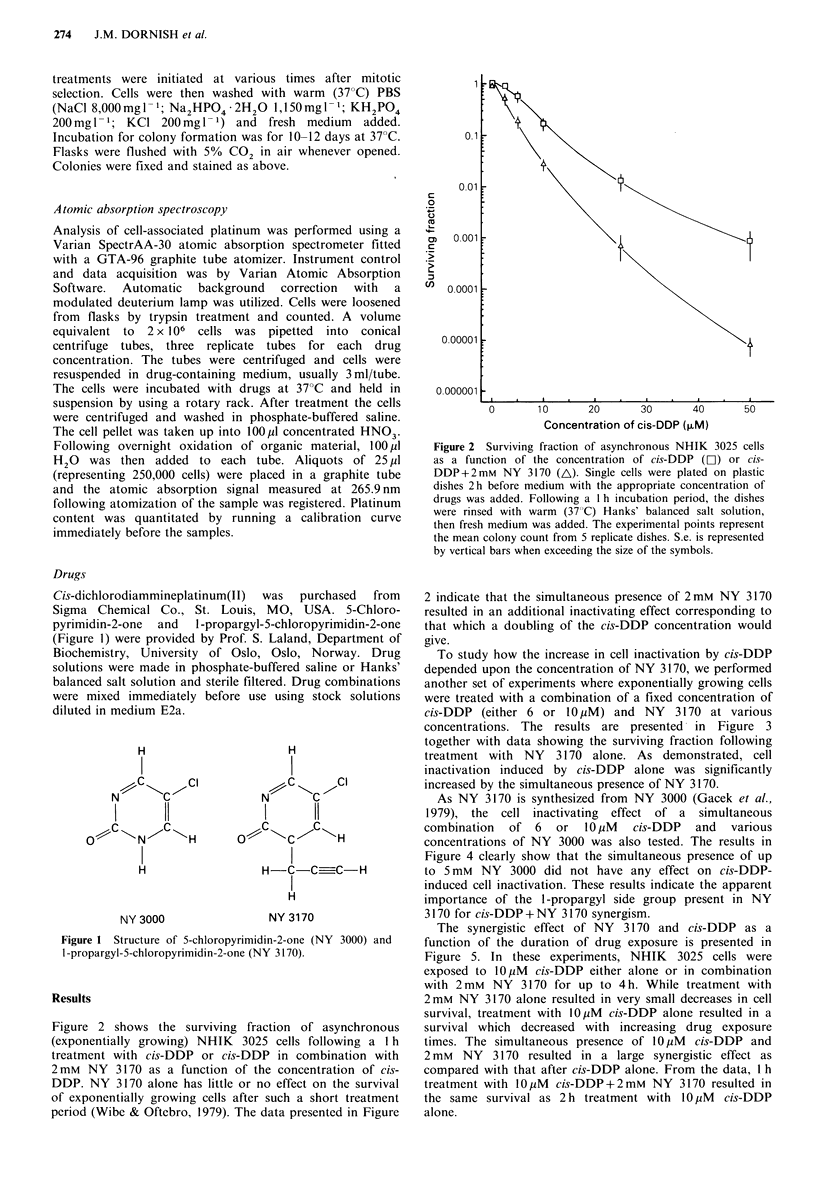

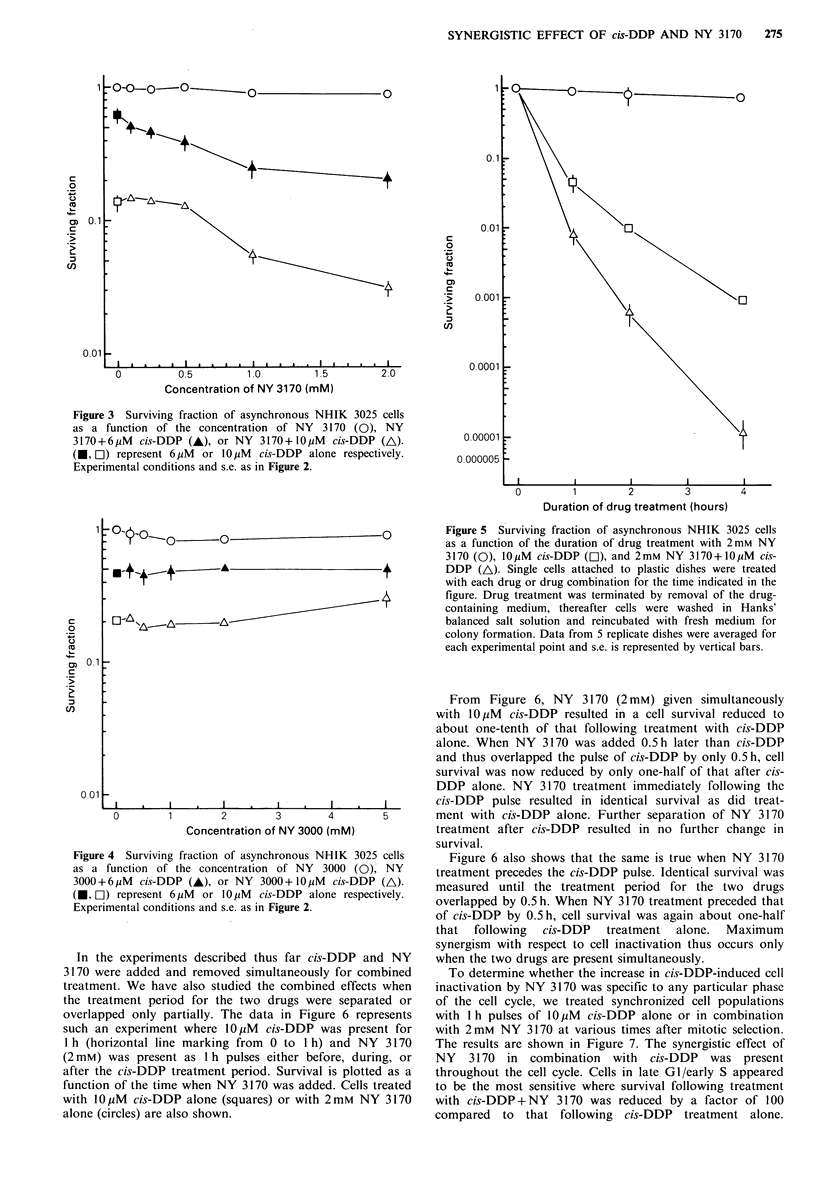

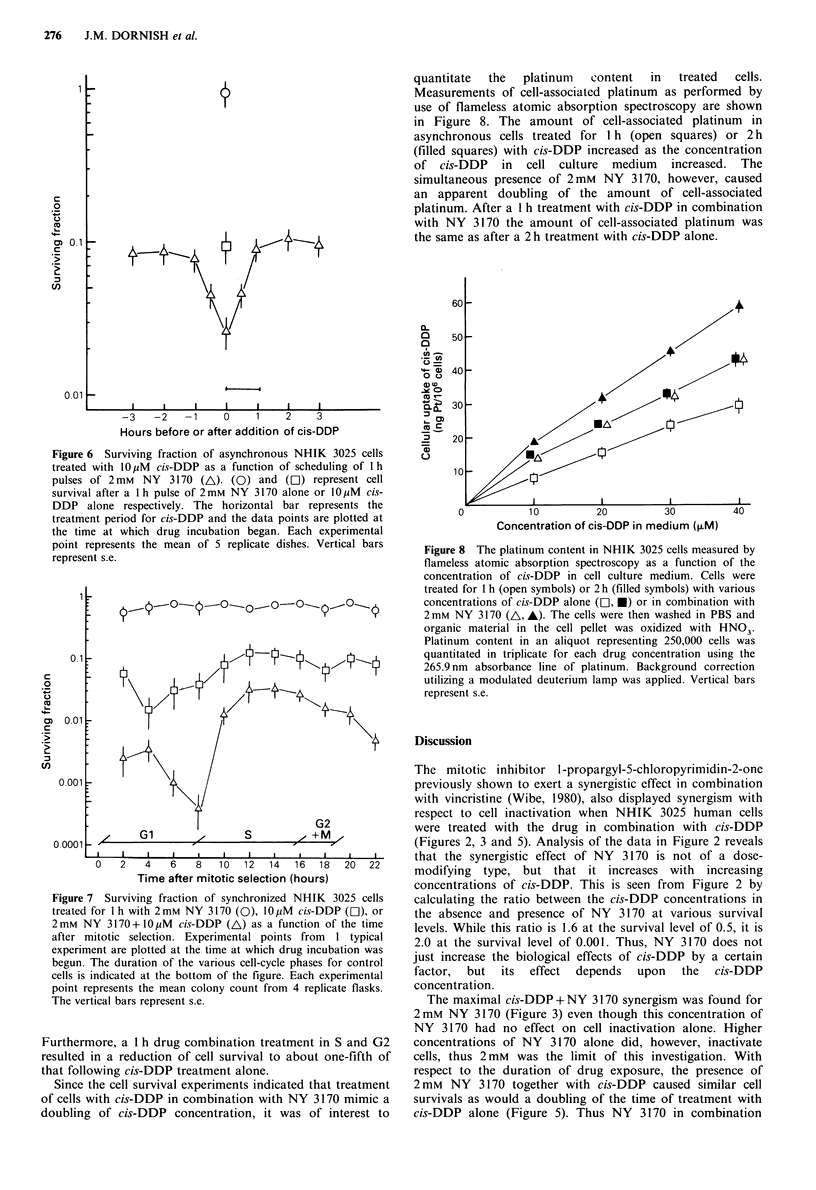

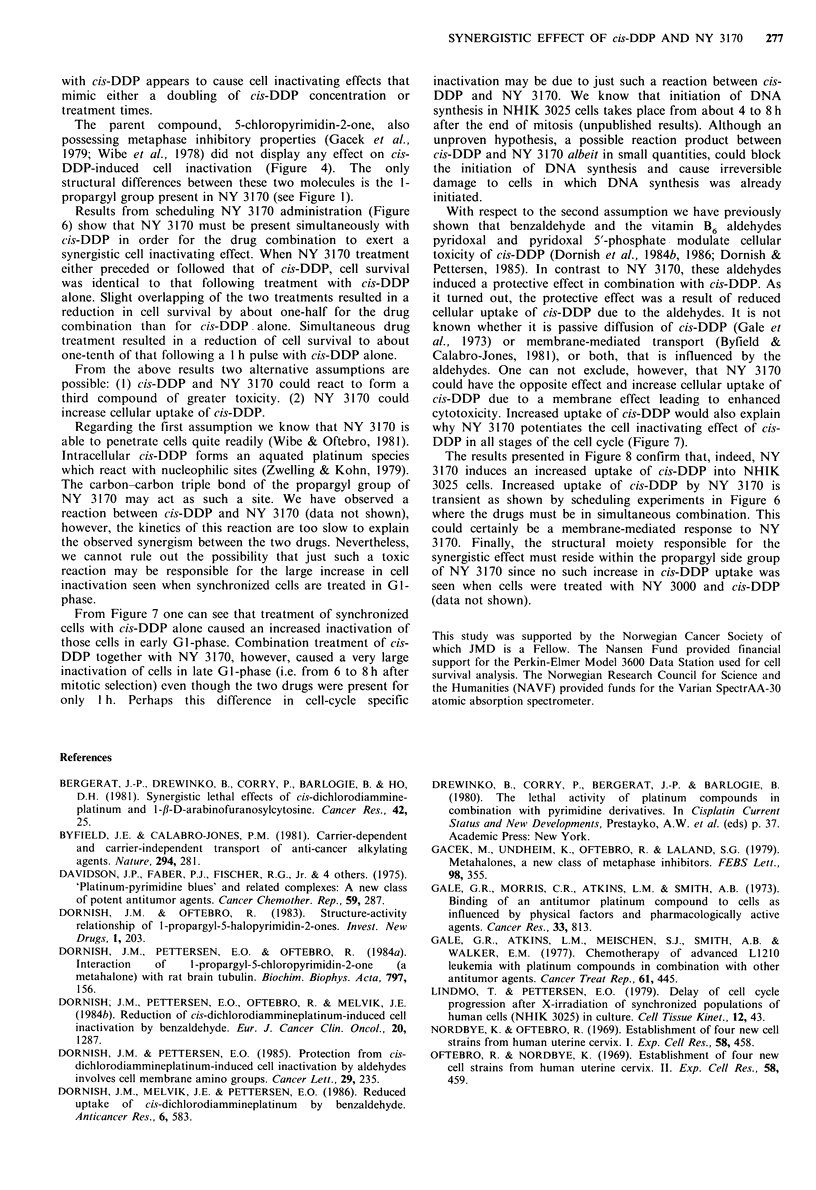

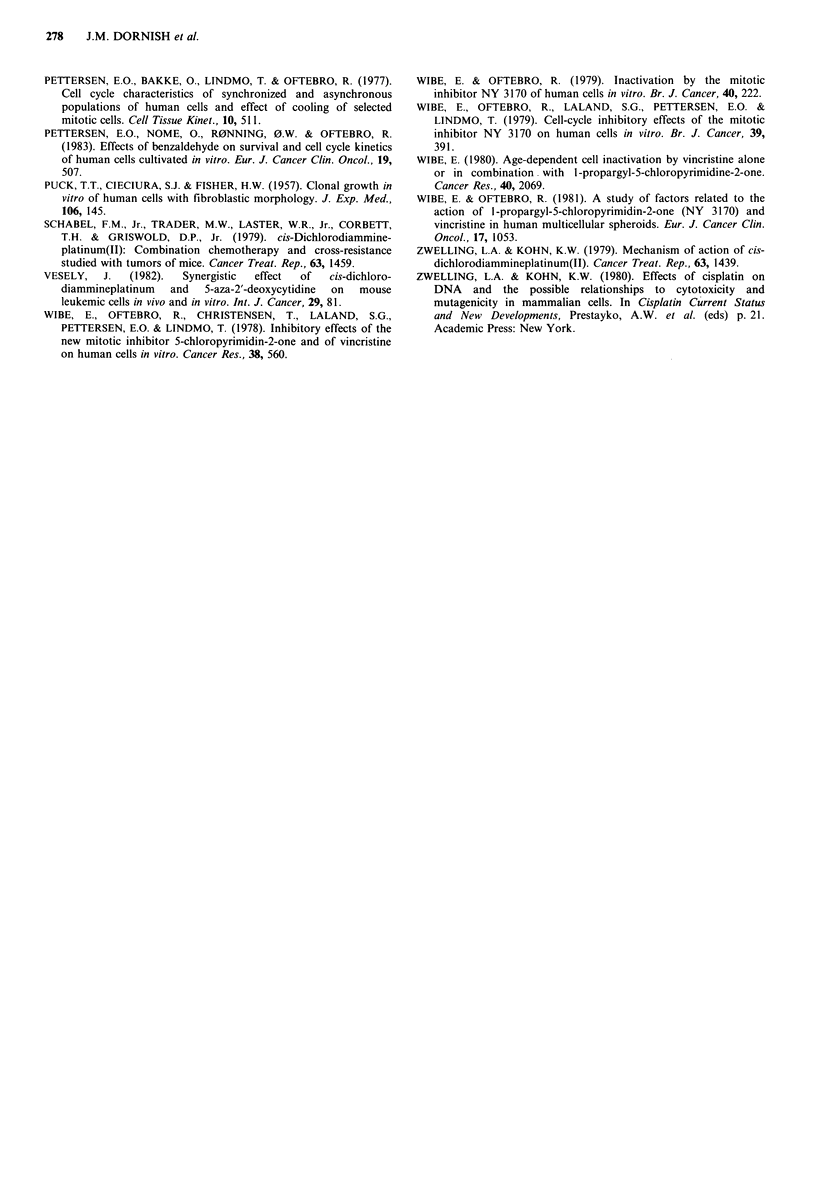


## References

[OCR_00699] Bergerat J. P., Drewinko B., Corry P., Barlogie B., Ho D. H. (1981). Synergistic lethal effect of cis-dichlorodiammineplatinum and 1-beta-D-arabinofuranosylcytosine.. Cancer Res.

[OCR_00705] Byfield J. E., Calabro-Jones P. M. (1981). Carrier-dependent and carrier-independent transport of anti-cancer alkylating agents.. Nature.

[OCR_00710] Davidson J. P., Faber P. J., Fischer R. G., Mansy S., Peresie H. J., Rosenberg B., VanCamp L. (1975). "Platinum-pyrimidine blues" and related complexes: a new class of potent antitumor agents.. Cancer Chemother Rep.

[OCR_00737] Dornish J. M., Melvik J. E., Pettersen E. O. (1986). Reduced cellular uptake of cis-dichlorodiammine-platinum by benzaldehyde.. Anticancer Res.

[OCR_00715] Dornish J. M., Oftebro R. (1983). Structure-activity relationship of 1-propargyl-5-halopyrimidin-2-ones. Metaphase arresting properties and competitive inhibition of colchicine binding to tubulin.. Invest New Drugs.

[OCR_00720] Dornish J. M., Pettersen E. O., Oftebro R. (1984). Interaction of 1-propargyl-5-chloropyrimidin-2-one (a metahalone) with rat brain tubulin.. Biochim Biophys Acta.

[OCR_00726] Dornish J. M., Pettersen E. O., Oftebro R., Melvik J. E. (1984). Reduction of cis-dichlorodiammineplatinum-induced cell inactivation by benzaldehyde.. Eur J Cancer Clin Oncol.

[OCR_00732] Dornish J. M., Pettersen E. O. (1985). Protection from cis-dichlorodiammineplatinum-induced cell inactivation by aldehydes involves cell membrane amino groups.. Cancer Lett.

[OCR_00749] Gacek M., Undheim K., Oftebro R., Laland S. G. (1979). Metahalones, a new class of metaphase inhibitors.. FEBS Lett.

[OCR_00760] Gale G. R., Atkins L. M., Meischen S. J., Smith A. B., Walker E. M. (1977). Chemotherapy of advanced L1210 leukemia with platinum compounds in combination with other antitumor agents.. Cancer Treat Rep.

[OCR_00754] Gale G. R., Morris C. R., Atkins L. M., Smith A. B. (1973). Binding of an antitumor platinum compound to cells as influenced by physical factors and pharmacologically active agents.. Cancer Res.

[OCR_00766] Lindmo T., Pettersen E. O. (1979). Delay of cell cycle progression after X-irradiation of synchronized populations of human cells (NHIK 3025) in culture.. Cell Tissue Kinet.

[OCR_00794] PUCK T. T., CIECIURA S. J., FISHER H. W. (1957). Clonal growth in vitro of human cells with fibroblastic morphology; comparison of growth and genetic characteristics of single epithelioid and fibroblast-like cells from a variety of human organs.. J Exp Med.

[OCR_00782] Pettersen E. O., Bakke O., Lindmo T., Oftebro R. (1977). Cell cycle characteristics of synchronized and asynchronous populations of human cells and effect of cooling of selected mitotic cells.. Cell Tissue Kinet.

[OCR_00788] Pettersen E. O., Nome O., Rønning O. W., Oftebro R. (1983). Effects of benzaldehyde on survival and cell-cycle kinetics of human cells cultivated in vitro.. Eur J Cancer Clin Oncol.

[OCR_00799] Schabel F. M., Trader M. W., Laster W. R., Corbett T. H., Griswold D. P. (1979). cis-Dichlorodiammineplatinum(II): combination chemotherapy and cross-resistance studies with tumors of mice.. Cancer Treat Rep.

[OCR_00805] Veselý J. (1982). Synergistic effect of cis-dichlorodiammineplatinum and 5-aza-2'-deoxycytidine on mouse leukemic cells in vivo and in vitro.. Int J Cancer.

[OCR_00825] Wibe E. (1980). Age-dependent cell inactivation by vincristine alone or in combination with 1-propargyl-5-chloropyrimidin-2-one.. Cancer Res.

[OCR_00830] Wibe E., Oftebro R. (1981). A study of factors related to the action of 1-propargyl-5-chloropyrimidin-2-one (NY 3170) and vincristine in human multicellular spheroids.. Eur J Cancer Clin Oncol.

[OCR_00810] Wibe E., Oftebro R., Christensen T., Laland S. G., Pettersen E. O., Lindmo T. (1978). Inhibitory effects of the new mitotic inhibitor 5-chloropyrimidin-2-one and of vincristine on human cells in vitro.. Cancer Res.

[OCR_00816] Wibe E., Oftebro R. (1979). Inactivation by the mitotic inhibitor NY 3170 of human cells in vitro.. Br J Cancer.

[OCR_00819] Wibe E., Oftebro R., Laland S. G., Pettersen E. O., Lindmo T. (1979). Cell-cycle inhibitory effects of the mitotic inhibitor NY 3170 on human cells in vitro.. Br J Cancer.

[OCR_00836] Zwelling L. A., Kohn K. W. (1979). Mechanism of action of cis-dichlorodiammineplatinum(II).. Cancer Treat Rep.

